# Supplementary material for: Content-rich biological network constructed by mining PubMed abstracts
Source: BMC Bioinformatics. 2004 Oct 8;5:147. doi: 10.1186/1471-2105-5-147 (PMC528731; doi:10.1186/1471-2105-5-147)
Supplement: Additional File 2 — The original results of the above study (non-essential files are deleted to keep the file size under the limit set by BMC bioinformatics). [file 1471-2105-5-147-S2.bz2 › chilibotAdditionalFile2/dip05/115ID8645696E459/html/ATPH_ATPG.html]

 


 **ATPH** and **ATPG** 
  
Found 9 abstracts in PubMed, retrieved 05.  
 

 What does Google say? 
 PDF only 
| .edu only 

---

**Interactive relationship** (e.g. stimulation, inhibition, etc)

**Non-interactive relationship** (e.g. studied together, co-existance, homology, etc.)

- Moreover, phytanic acid activates brown adipocyte differentiation long term exposure of brown preadipocytes to phytanic acid promoted the acquisition of the brown adipocyte morphology and caused a co ordinate induction of the mRNAs for gene markers of brown adipocyte differentiation, such as UCP1, adipocyte lipid binding protein  [ **ATPH** ]  aP2, lipoprotein lipase, the glucose transporter GLUT4 or subunit II  [ **ATPG** ]  of cytochrome c oxidase.  Ref: 11829740 Biochem J, 2002
- A single 4.5 kb transcript was detected with  **atpG** , F, D and A gene probes that could span the whole operon, but no transcript could be detected with atpI and  **atpH**  probes.  Ref: 1404401 J Mol Biol, 1992
- The C. thermoaceticum atp operon contained nine genes arranged in the order atpI i, atpB a, atpE c, atpF b,  **atpH**  delta, atpA alpha,  **atpG**  gamma, atpD beta, and atpC epsilon.  Ref: 9171425 J Bacteriol, 1997
- Eight genes atpI, atpB, atpE 1, atpE 2, atpE 3, atpF,  **atpH** , and atpA upstream of and contiguous with the previously described genes  **atpG** , atpD, and atpC were cloned from chromosomal DNA of Acetobacterium woodii.  Ref: 10567365 J Biol Chem, 1999
